# A Rare Case of Gastrointestinal Stromal Tumor of the Abdominal Cavity: A Case Report

**DOI:** 10.7759/cureus.44926

**Published:** 2023-09-08

**Authors:** Carlos Ignacio Rafael-Perez, Alexis Jared Paz-López, Paola Saskia Castañeda-Anaya

**Affiliations:** 1 Digestive and Endocrine Surgery Department, Unidad Médica de Alta Especialidad No. 25, Monterrey, MEX

**Keywords:** exploratory laporotomy, digestive surgery, surgical oncologist, tyrosine kinase receptor inhibitors, gastrointestinal stromal tumors (gists)

## Abstract

Gastrointestinal stromal tumors (GISTs) are rare gastrointestinal neoplasms. We are presenting a 57-year-old female patient with a GIST of considerable dimensions that was deemed unresectable during the initial intervention. The patient had a previous surgical record of a peritoneal sarcomatosis tumor that covered approximately 50% of the abdominal cavity. The patient underwent surgical procedures, including exploratory laparotomy, lumpectomy, splenectomy, proximal gastrectomy, esophagogastric anastomosis, pyloroplasty, jejunostomy, and left diaphragm plasty. One of the observed results was the presence of a 50×40 cm exophytic multilobed cerebroid-like tumor in the abdomen region, specifically dependent on the gastric fundus. The primary treatment for patients without metastases is surgical removal of the tumor, with wedge resection being recommended for the preservation of organ function and quality of life postoperatively.

## Introduction

Gastrointestinal stromal tumors (GISTs) are uncommon, accounting for only 1% to 3% of gastrointestinal neoplasms, and are sarcomas derived from interstitial Cajal cell precursors [1,2]. The average age of diagnosis is estimated to be in the sixth decade, and it occurs frequently in 6.8 to 14.5 cases per million people, with a higher incidence in the stomach (51%), small intestine (36%), colon (7%), rectum (5%), and esophagus (1%) [3,4]. Spindle cell type (70%), epithelial cell type (20%), and mixed type (10%) are the primary histological subtypes of GIST. Most GISTs devoid of subepithelial lesions result from oncogenic mutations in the KIT receptor tyrosine kinase and/or platelet-derived growth factor receptor (PDGFR) [5].

Eighteen percent of patients are asymptomatic at the time of diagnosis and are typically discovered during endoscopies or other surgical procedures [6,7]. Patients report gastrointestinal bleeding, manifested by melena or hematemesis, as well as weakness, abdominal distension, and distress caused by the tumor's mass effect [8].

This report pertains to a patient presenting with a GIST of considerable dimensions that was deemed unresectable during the initial intervention.

## Case presentation

Patient information

In this case report, we present a clinical case of a 57-year-old female patient who does not exhibit any notable chronic degenerative disorders. The patient had stomach pain and a gradual enlargement of the abdomen one year ago. This was accompanied by difficulties in consuming food orally, feelings of weakness and fatigue, reduced appetite, and an unintentional weight reduction of around 12 kg, without any specific dietary or exercise regimen.

Due to the manifestation of these symptoms, the patient decided to pursue medical attention in a general hospital, initiating her surgical treatment regimen. A computed tomography (CT) scan of the abdomen and pelvis was arranged to investigate the presence of a tumor mass in the left hypochondrium, with the aim of excluding the possibility of an exophytic stromal tumor originating from the stomach. After the passage of one month, an endoscopy was conducted and during this procedure, a rounded submucosal lesion of approximately 12×10 cm, characterized by its firm texture, was observed in the gastric fundus. As part of the examination, a sample was obtained from the lesion. The biopsy report indicated the presence of moderate chronic peptic gastritis and spindle cell mesenchymal neoplasm. The protocol is continued by sending it to the general surgery service. The medical practitioners decided to conduct an exploratory laparotomy procedure, during which they observed the existence of a peritoneal sarcomatosis tumor that encompassed almost 50% of the abdominal cavity. The tumor demonstrated a state of increased vascularity and was observed to be strongly attached to adjacent organs, hence posing challenges in its surgical removal. Following the unsuccessful surgical operation, the patient was then sent to a tertiary care center to undergo further examination and explore the possibility of an alternate surgical regimen.

Diagnostic assessment

**Figure 1:**
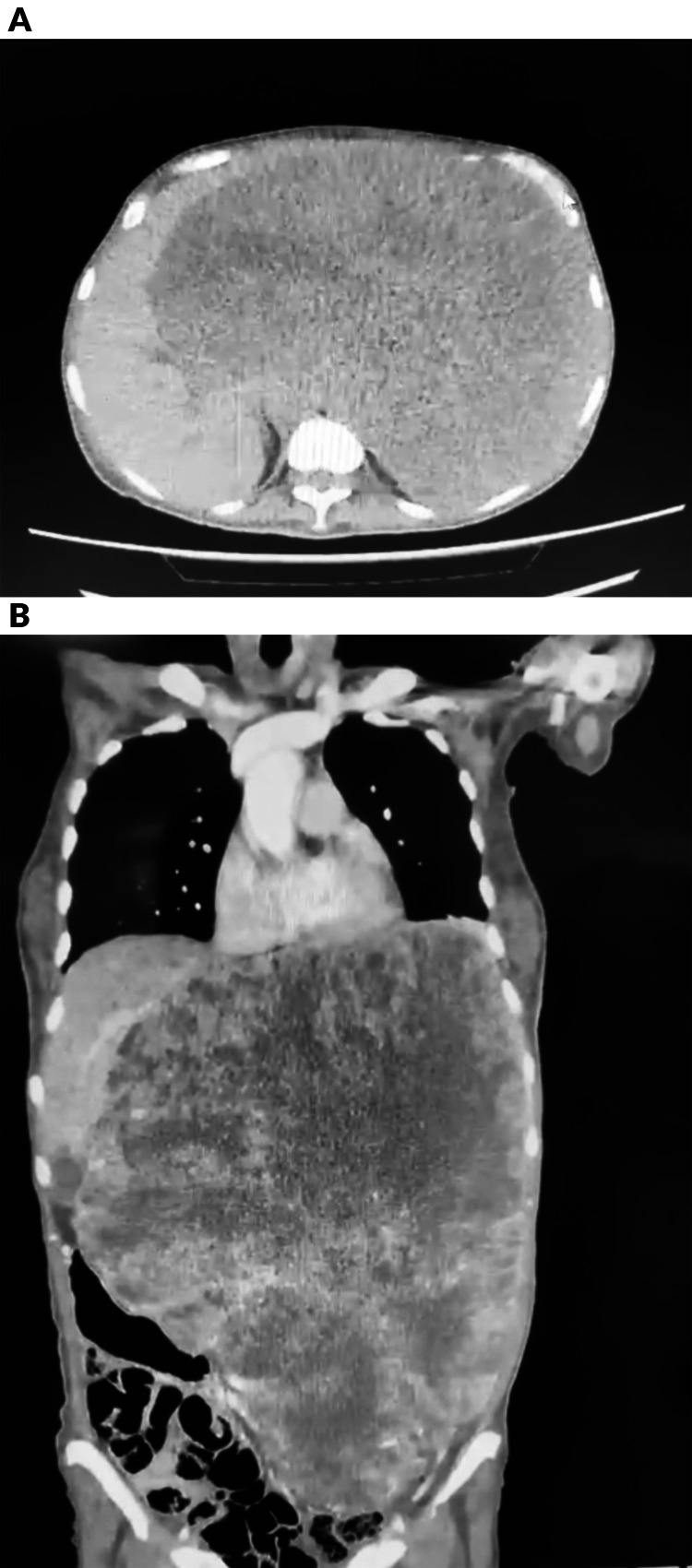
A. Axial view of the abdominal CT scan where an irregularly shaped tumor is observed, not delimited on the edges of the stomach. B. Coronal view of abdominal CT scan where it is observed that the tumor exerts a significant mass effect on adjacent structures, displacing them contralaterally, preserving the interface. The tumor measures 248x154x345 mm.

A new CT scan of the patient reveals the absence of well-defined boundaries of the stomach, indicating the presence of an irregularly shaped tumor. The tumor exhibits clearly demarcated edges and displays a heterogeneous appearance with areas of decreased density, which may suggest tissue necrosis. Additionally, the tumor exerts pressure on surrounding structures, and its dimensions are measured at 248x154x345 mm, with a calculated volume of 6983 mL (Figure 1). The laboratory findings of the patient can be found in Table 1. 

**Table 1:** Patient's laboratory results.

	Patient's results	Normal range
Leukocyte count	9.6 K/uL	4.5-11.0 K/uL
Hemoglobin level	12.4 g/dL	12.0-16.0 g/dL
Hematocrit value	39.10%	36.0-46.0%
Platelet count	346 K/uL	130-400x103/uL
Glucose concentration	76 mg/dL	70-110 mg/dL
Urea level	29.9 mg/dL	6-24 mg/dL
Blood urea nitrogen level	14 mg/dL	8-25 mg/dL
Creatinine concentration	0.5 mg/dL	0.6-1.1 mg/dL
Total bilirubin level	0.8 mg/dL	0.0-1.0 mg/dL
Lactic dehydrogenase activity	383 u/L	105-333 u/L
Albumin concentration	3.5 g/dL	3.5-5.4 g/dL

Therapeutic intervention

The decision was made to proceed with scheduling the patient for surgery, during which a series of procedures were conducted, including an exploratory laparotomy, tumorectomy, splenectomy, proximal gastrectomy, esophagogastric anastomosis, pyloroplasty, jejunostomy, and left diaphragm plasty.

The surgical procedure commences by making an incision along the suprainfraumbilical midline, revealing a sizable tumor. A dissection is then carried out to detach the tumor from the peritoneum, followed by a circumferential dissection to separate any adhesions to neighboring organs. These organs are displaced and separated from the colon and omentum. Finally, the pancreatic edge is ligated. The splenic vessels, which encompass the spleen, the short gastric vessels, the left gastroepiploic vessels, and the left gastric vessels are ligated. Additionally, the pars flaccida is dissected in the direction of the esophageal hiatus. The dissection of the hiatus has been successfully carried out, resulting in the cutting of the esophagus.

Subsequently, the diaphragm cuff has been performed, allowing for the extraction of the piece as a whole. Following this, diaphragm plasty was conducted, along with pylorotomy and anastomosis. To facilitate drainage, a Blake 19 French retroanastomotic drain has been inserted. Additionally, Tisseel and Gelfoam have been placed in the wound. To ensure proper nutrition, a feeding jejunostomy has been created. Furthermore, a left endopleural tube has been inserted, and the closure has been carried out using appropriate planes.

The surgical procedure lasted for eight hours, during which a total blood loss of 3600 mL was observed. To address this, the patient required the administration of eight units of packed red blood cells, five units of fresh frozen plasma, and platelet apheresis. The patient additionally necessitated the administration of 10 ampules of hCO3, 1 mg of sulfate, 5 g of calcium gluconate, 20 mg of furosemide, 2 g of cefotaxime intravenously, and 8 mg of dexamethasone intravenously.

**Figure 2:**
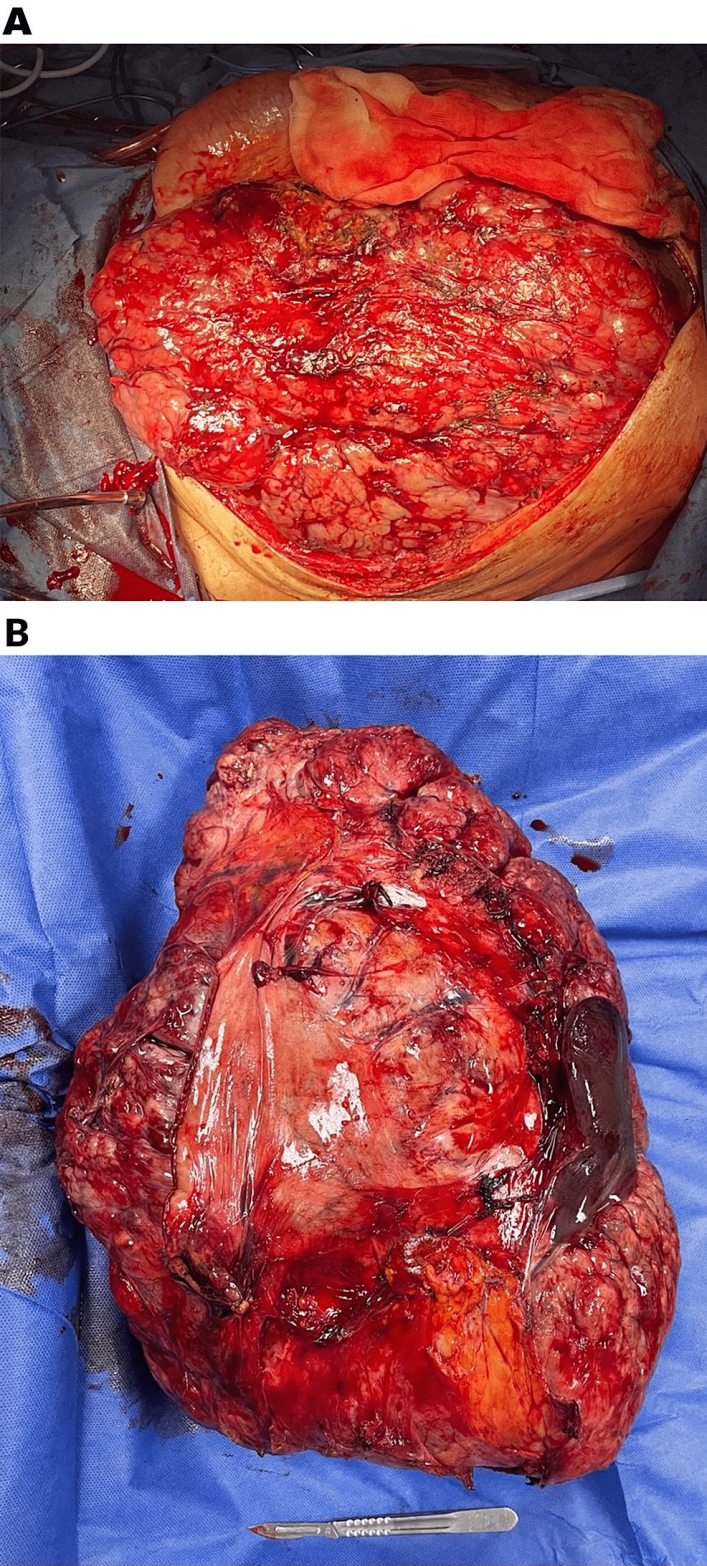
A. Tumor in the abdominal cavity dependent on the stomach. B. Resected cerebroid-like tumor.

**Figure 3:**
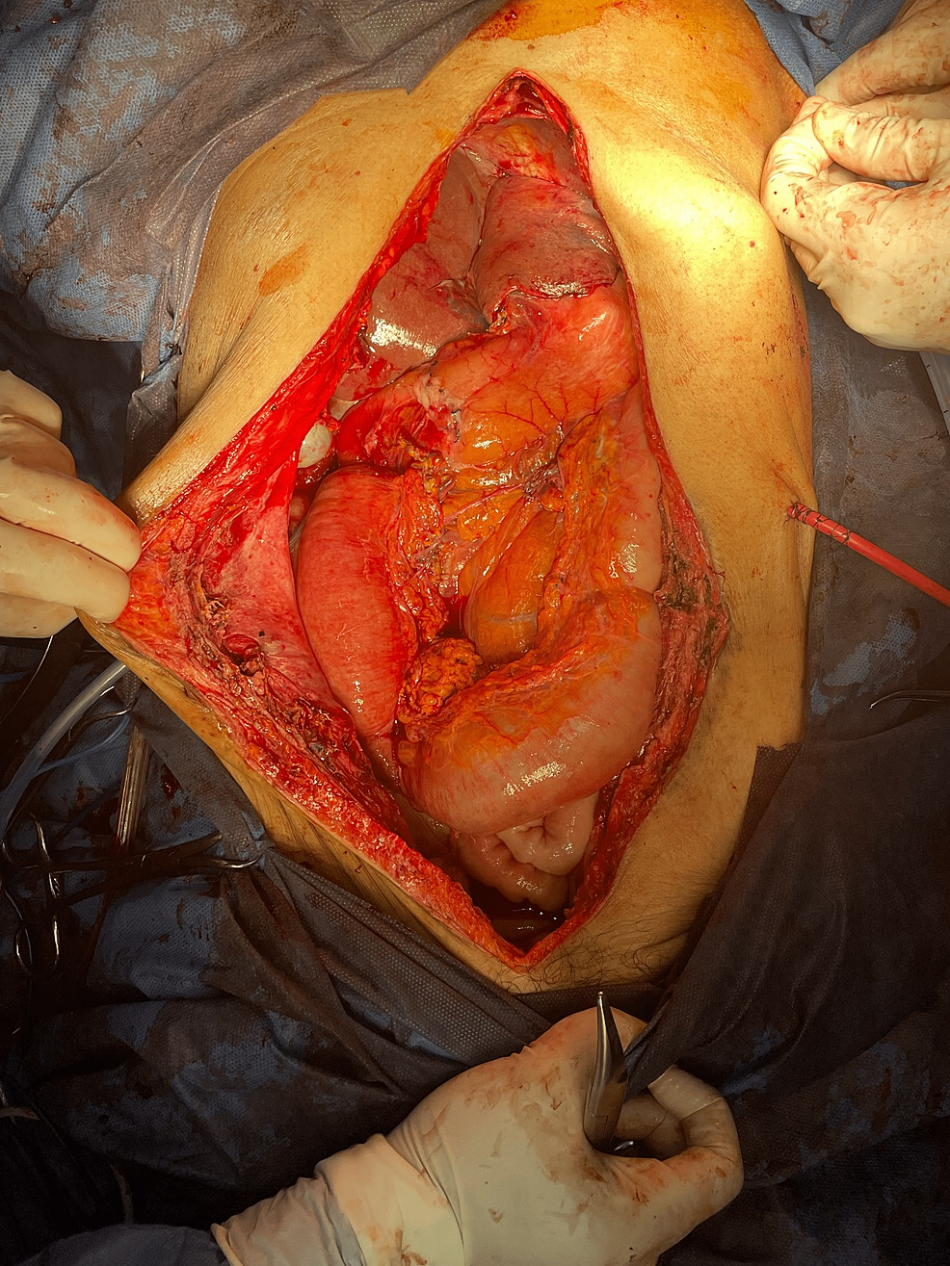
Abdominal cavity after surgical removal of the tumor.

Following the surgical procedure, the medical team identified the existence of a tumor in the abdominal region measuring 50x40 cm. This tumor exhibited an exophytic growth pattern, displaying many lobes resembling the structure of the cerebrum. Notably, the tumor was mostly attached to the gastric fundus. The tumor exhibited displacement of multiple abdominal structures, such as the omentum, liver, spleen, and left diaphragm (Figure 2). Figure 3 illustrates the presence of the abdominal cavity after surgical removal of the tumor.

After the completion of the surgical intervention, a clinical decision was taken to relocate the patient to the intensive care unit (ICU) because of the diagnoses of grade IV hemorrhagic shock, suspected gastric GIST, and hydroelectrolyte imbalance attributed to mild hypokalemia. The individual had lingering signs of sedation. The administration of dexmedetomidine was initiated at a dosage of 0.4 mcg/kg/hr, resulting in a Richmond Agitation-Sedation Scale score of zero and a low ventilator minute volume index in volume-controlled continuous mandatory ventilation. The patient's parameters include a positive end-expiratory pressure (PEEP) of 5 cmH_2_O, tidal volume (VT) of 320 mL, respiratory rate (RR) of 16 breaths per minute, inspiratory flow of 30 L/min, PEEP of 5 cmH_2_O, trigger sensitivity of 2 L/min, fraction of inspired oxygen (FiO_2_) of 40%, peak inspiratory pressure of 17 cmH_2_O, minute ventilation of 6.5 L/min, and inspiratory-expiratory ratio of 1:3. The patient is attaining oxygen saturation levels above 95%. Hemodynamically, the patient is receiving norepinephrine at a rate of 0.06 mcg/kg/min to maintain mean arterial pressure (MAP) within the target range of 70-80 mmHg. The norepinephrine infusion is gradually being reduced until it is discontinued. The patient's capillary refill time is measured at 2-3 s. Upon the patient's recovery from sedation, the ventilatory modality was transitioned to continuous positive airway pressure (CPAP) with specific settings (PEEP 4, PSOP 0, FiO_2_ 35%). The patient exhibited satisfactory tolerance to this change, leading to the withdrawal of norepinephrine as the MAP reached the desired targets. Subsequently, tests were conducted to assess the readiness for mechanical ventilation withdrawal, which indicated a rapid superficial respiration index of 25. Based on this information, a decision was made to extubate the patient approximately four hours later.

Throughout the patient's duration in the ICU, the patient maintained neurological integrity and hemodynamic stability. Telemetry monitoring revealed tachycardia from the time of admission. Additionally, her blood pressure was dependent on the administration of the norepinephrine vasopressor, with a maximum dosage of 0.22mcg/mn/hour. The process of weaning is conducted while maintaining a positive airway pressure of 65 mmHg. The endopleural tube exhibited a drainage volume of 1200cc over a span of 72 hours, characterized by serohematic fluid. Additionally, the abdomen was fitted with a Blake drainage system, which reported a drainage volume of 160cc during a 24 hour period, also characterized by serohematic fluid. Notably, the drainage volumes exhibited a steady reduction over time. The administration of jejunostomy feeding was initiated using a 5% glucose solution, which was well-tolerated. Subsequently, the patient was admitted to the general hospital and was discharged 10 days following the surgical procedure, exhibiting satisfactory post-surgery progress.

**Figure 4:**
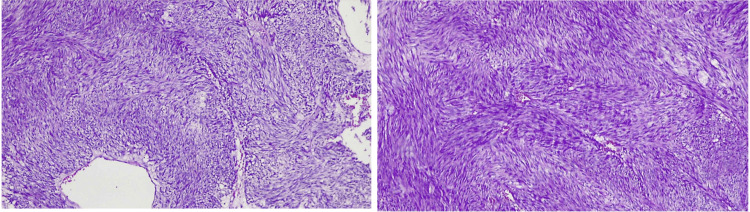
Histopathology sample where high mitotic content is observed, up to 20 mitoses per 5 square millimeters, with areas of necrosis.

The surgical material was submitted for histopathological analysis, revealing the presence of a spindle cell GIST. The tumor had a mitotic index of 20 mitoses per 5 square millimeters, indicating an increased rate of cell division. Based on its histological characteristics, the tumor was classified as grade G2, indicating moderate differentiation. Additionally, the tumor was considered high risk due to its size exceeding 5 cm (Figure 4).

Follow-up and outcomes

The patient attended a subsequent appointment to follow up on the oncology consultation. During this visit, medical management commenced by prescribing a daily oral intake of imatinib at a dosage of 400 mg for three years. Currently, four months post-surgery, the patient is asymptomatic and is being monitored by medical oncologists.

## Discussion

GISTs are characterized by a very low occurrence rate and typically exhibit dimensions ranging from 2 to 6 cm [9,10]. The largest GIST documented in existing literature was described by Mohamed et al., measuring 42x31 cm [11]. However, the present case concerns a patient who presented an exceptionally sizable tumor spanning 50x40 cm, which encompasses about 50% of the patient's abdominal cavity.

The preferred treatment for patients without metastases is surgical removal of the tumor. The primary objective is to attain an R0 resection, and it is advisable to consider a wedge resection that ensures the preservation of organ function and the maintenance of a satisfactory quality of life postoperatively. The patient underwent two surgical procedures, with the latter involving the intervention of multiple organs and subsequent monitoring in an intensive care setting. Ensuring appropriate surgical protocol is of utmost importance, taking into account the capabilities of the surgical staff and the intrahospital resources, in order to mitigate potential risks and safeguard the well-being of patients.

Tyrosine kinase inhibitors are recommended for individuals who have unresectable or recurring malignancies or the presence of metastases [12,13]. Imatinib, a tyrosine kinase inhibitor, has been established as the primary treatment option. It has demonstrated efficacy in increasing the duration of recurrence-free life following surgery or prolonging overall survival in cases where surgical removal is not feasible or when metastasis has occurred [14,15]. Additionally, there exist second- and third-line therapeutic options, namely sunitinib and regorafenib, that can be employed in cases of advanced disease where treatment with imatinib proves ineffective [14]. Other strategies for addressing GISTs such as nutrition enriched with supplements like glutamine or omega 3 have been studied, without reporting significant results [16]. As of this case, the patient has continued to receive imatinib treatment, resulting in a positive clinical progression and alleviation of symptoms after the surgical intervention.

Prior studies have shown that individuals with operable tumors have achieved a median survival duration of up to 15 years without tumor recurrence, with a minority of patients encountering recurrence a decade following the surgical intervention [17]. Nevertheless, it is suggested that patients receive tyrosine kinase inhibitors for 12 months as a post-surgical follow-up treatment [10].

## Conclusions

GISTs are characterized by their low incidence rates, often resulting in incidental diagnoses among most patients. The significance of this condition resides in the timely identification, as it contributes to enhancing patient survival. In the present scenario, a patient exhibiting symptoms suggestive of GIST is seen, for whom the preferred treatment approach is R0 resection. It is imperative to bear in mind that surgical resection represents the benchmark approach for addressing these tumors. Subsequently, chemotherapy is administered, and long-term monitoring is conducted by medical oncologists.

## References

[1] Al-Share B, Alloghbi A, Al Hallak MN (2021). Gastrointestinal stromal tumor: a review of current and emerging therapies. Cancer Metastasis Rev.

[2] Serrano C, George S (2020). Gastrointestinal stromal tumor: challenges and opportunities for a new decade. Clin Cancer Res.

[3] Tran T, Davila JA, El-Serag HB (2005). The epidemiology of malignant gastrointestinal stromal tumors: an analysis of 1,458 cases from 1992 to 2000. Am J Gastroenterol.

[4] Tzen CY, Wang JH, Huang YJ (2007). Incidence of gastrointestinal stromal tumor: a retrospective study based on immunohistochemical and mutational analyses. Dig Dis Sci.

[5] Hirota S, Isozaki K, Moriyama Y (1998). Gain-of-function mutations of c-kit in human gastrointestinal stromal tumors. Science.

[6] Scherübl H, Faiss S, Knoefel WT, Wardelmann E (2014). Management of early asymptomatic gastrointestinal stromal tumors of the stomach. World J Gastrointest Endosc.

[7] Søreide K, Sandvik OM, Søreide JA, Giljaca V, Jureckova A, Bulusu VR (2016). Global epidemiology of gastrointestinal stromal tumours (GIST): a systematic review of population-based cohort studies. Cancer Epidemiol.

[8] Joensuu H, Hohenberger P, Corless CL (2013). Gastrointestinal stromal tumour. Lancet.

[9] Gheorghe G, Bacalbasa N, Ceobanu G (2021). Gastrointestinal stromal tumors - a mini review. J Pers Med.

[10] Mantese G (2019). Gastrointestinal stromal tumor: epidemiology, diagnosis, and treatment. Curr Opin Gastroenterol.

[11] Mohamed A, Botros Y, Hanna P, Lee S, Baddoura W, Zuberi J, Damani T (2018). Gigantic GIST: a case of the largest gastrointestinal stromal tumor found to date. Case Rep Surg.

[12] Li J, Ye Y, Wang J (2017). Chinese consensus guidelines for diagnosis and management of gastrointestinal stromal tumor. Chin J Cancer Res.

[13] Nishida T, Hirota S, Yanagisawa A (2008). Clinical practice guidelines for gastrointestinal stromal tumor (GIST) in Japan: English version. Int J Clin Oncol.

[14] Joensuu H, Eriksson M, Sundby Hall K (2012). One vs three years of adjuvant imatinib for operable gastrointestinal stromal tumor: a randomized trial. JAMA.

[15] Verweij J, Casali PG, Zalcberg J (2004). Progression-free survival in gastrointestinal stromal tumours with high-dose imatinib: randomised trial. Lancet.

[16] Ma C, Tsai H, Su W, Sun L, Shih Y, Wang J (2018). Combination of arginine, glutamine, and omega-3 fatty acid supplements for perioperative enteral nutrition in surgical patients with gastric adenocarcinoma or gastrointestinal stromal tumor (GIST): a prospective, randomized, double-blind study. J Postgrad Med.

[17] Joensuu H, Vehtari A, Riihimäki J (2012). Risk of recurrence of gastrointestinal stromal tumour after surgery: an analysis of pooled population-based cohorts. Lancet Oncol.

